# PISTACHIo (PreemptIon of diSrupTive behAvior in CHIldren): real-time monitoring of sleep and behavior of children 3–7 years old receiving parent–child interaction therapy augment with artificial intelligence — the study protocol, pilot study

**DOI:** 10.1186/s40814-023-01254-w

**Published:** 2023-02-09

**Authors:** Maria Saliba, Noelle Drapeau, Michelle Skime, Xin Hu, Carolyn Jonas Accardi, Arjun P. Athreya, Jacek Kolacz, Julia Shekunov, Dean P. Jones, Paul E. Croarkin, Magdalena Romanowicz

**Affiliations:** 1 Department of Psychiatry and Psychology, Mayo Clinic, Rochester, MN 55905 USA; 2 Department of Pediatrics, Mayo Clinic, Rochester, MN 55905 USA; 3 Clinical Biomarkers Laboratory, Division of Pulmonary, Allergy, Critical Care and Sleep Medicine, Department of Medicine, Emory University, Atlanta, GA 30322 USA; 4 Department of Molecular Pharmacology and Experimental Therapeutics, Mayo Clinic, Rochester, MN 55905 USA; 5 Department of Psychiatry and Behavioral Health, The Ohio State University Wexner Medical Center, Columbus, OH 43210 USA

**Keywords:** Artificial intelligence, Parent–child interaction therapy, Disruptive behaviors

## Abstract

**Background:**

Emotional behavior problems (EBP) are the most common and persistent mental health issues in early childhood. Early intervention programs are crucial in helping children with EBP. Parent–child interaction therapy (PCIT) is an evidence-based therapy designed to address personal difficulties of parent–child dyads as well as reduce externalizing behaviors. In clinical practice, parents consistently struggle to provide accurate characterizations of EBP symptoms (number, timing of tantrums, precipitating events) even from the week before in their young children. The main aim of the study is to evaluate feasibility of the use of smartwatches in children aged 3–7 years with EBP.

**Methods:**

This randomized double-blind controlled study aims to recruit a total of 100 participants, consisting of 50 children aged 3–7 years with an EBP measure rated above the clinically significant range (T-score ≥ 60) (Eyberg Child Behavior Inventory-ECBI; Eyberg & Pincus, 1999) and their parents who are at least 18 years old. Participants are randomly assigned to the artificial intelligence-PCIT group (AI-PCIT) or the PCIT-sham biometric group. Outcome parameters include weekly ECBI and Pediatric Sleep Questionnaire (PSQ) as well as Child Behavior Checklist (CBCL) obtained weeks 1, 6, and 12 of the study. Two smartphone applications (Garmin connect and mEMA) and a wearable Garmin smartwatch are used collect the data to monitor step count, sleep, heart rate, and activity intensity. In the AI-PCIT group, the mEMA application will allow for the ecological momentary assessment (EMA) and will send behavioral alerts to the parent.

**Discussion:**

Real-time predictive technologies to engage patients rely on daily commitment on behalf of the participant and recurrent frequent smartphone notifications. Ecological momentary assessment (EMA) provides a way to digitally phenotype in-the-moment behavior and functioning of the parent–child dyad. One of the study’s goals is to determine if AI-PCIT outcomes are superior in comparison with standard PCIT. Overall, we believe that the PISTACHIo study will also be able to determine tolerability of smartwatches in children aged 3–7 with EBP and could participate in a fundamental shift from the traditional way of assessing and treating EBP to a more individualized treatment plan based on real-time information about the child’s behavior.

**Trial registration:**

The ongoing clinical trial study protocol conforms to the international Consolidated Standards of Reporting Trials (CONSORT) guidelines and is registered in clinicaltrials.gov (ID: NCT05077722), an international clinical trial registry.

## Introduction

Externalizing behaviors are the most common and persistent mental health issues in early childhood. It is estimated that nearly 25% of preschool-aged children struggle with psychosocial stress and social-emotional issues [1]. Between 15 and 34% of young children are reported to have significant early externalizing behavior problems (EBP) such as aggression, oppositional behaviors, problems with concentration, and impulsivity [2], as measured by ECBI (Eyberg Child Behavior Inventory) [3]. If EBP symptoms that emerge at a very young age are untreated, ongoing impairments persist throughout development and with ensuing negative outcomes such as academic and legal difficulties, violent behaviors and significant mental health issues, and substance abuse [4–6]. Disruptive behavior disorders and EBP in preschool are also the most common reason for referral to specialty mental health clinics [7]. Early intervention programs that are evidence-based and easy to implement are crucial in helping young children with EBP [8].

Children with externalizing problems often display both physical (fighting with peers, hitting, kicking) and emotional (struggles with angry and aggressive feelings) symptoms. Both physical and emotional displays distract them from their learning, as well as ability to make social connections. Children with EBP are less engaged in their relationship with their parents who mostly resort to nagging them about their behaviors and lack of compliance. Problems observed in daycare and preschool are often associated with poor academic performance. Externalizing behaviors distract from the ability to complete certain required academic tasks [9].

In clinical practice, parents consistently struggle to provide accurate characterizations of EBP symptoms (number, timing of tantrums, precipitating events) even from the week before in their young children. They also are frequently unsure how their child slept. Rating scales assist parents with the recall, but they are not always reliable. Child behavior often differs depending on the situation and setting. In addition, different informants may elicit different behaviors from the same child, they may differ in their opinions regarding what is considered a significant tantrum and what is not, and they also may interpret the same behavior in different ways [10]. Clinicians rely on a brief period of observation of the child in an artificial environment of their office that is not always reflective of the child’s behavior at home and/or school. These factors contribute to suboptimal outcomes in terms of diagnosis of young children emotional and behavioral problems. This is problematic as EBP is common, starts early in life, and can often lead to significant disabilities [11]. Our ongoing program of research, and the clinical trial described by these protocol manuscripts, proposes the use of wearable-augmented prediction technology, such as smartwatches coupled with artificial intelligence (AI) to predict onset of child’s disruptive behavior and foster better parenting practices. This in turn would enable the clinician to design an individualized treatment plan based on real-time information about the child’s behavior.

### The need for wearables in promoting parenting practices

PCIT (parent–child interaction therapy) is one of the four behavior parent training (BPT) programs that are considered “well supported by research evidence” by the California Evidence-Based Clearinghouse for Child Welfare [11]. It is a dyadic parent–child therapy technique that is unique due to its utilization of in vivo parent coaching via bug-in-the-ear treatment from a therapist behind one-way glass. Most importantly, PCIT allows for individually tailored coaching that can address personal difficulties of parent–child dyads. A recent meta-analysis of the effectiveness of PCIT, focused mainly on modifications, study design, and bias, showed that PCIT was superior not only in comparing case/control groups for reducing externalizing behaviors but also in helping with parent- and child-related stress [12]. The review additionally showed that PCIT effectiveness was not affected by session length, various settings (academic versus community), or presenting problems (disruptive behaviors alone as compared to other problems complicated by disruptive behaviors). Moreover, PCIT’s unique focus on goal criteria makes the components of PCIT ideal for modifications [12].

Despite PCIT effectiveness, high dropout rates (in some studies as high as 50%) prior to completion of the treatment remain a significant problem [13]. Currently, there are some efforts to reduce high attrition by the use of new technologies such as telehealth which remotely delivers real-time therapy to the patient’s home [14]. Another significant challenge lies in the ability to frequently engage and remind parents of the parenting practices taught during PCIT sessions [15]. The current study aims to examine and facilitate further development of an innovative artificial intelligence PCIT (AI-PCIT) protocol augmented with wearable technologies such as smartwatches.

Wearable technologies such as smartwatches allow for objectively measuring activities that extend beyond observations reported in the clinical settings. Wearables have been tested for continuous monitoring during pregnancy [16] and promoting physical activity in adolescents with juvenile idiopathic arthritis [17]. Despite the clinical need, wearable technologies have not been widely studied nor implemented for the treatment of young children with EBP.

### Objectives

The overall objectives of this AI-PCIT treatment clinical trial are to evaluate feasibility of the use of wearables such smartwatches: in children aged 3–7 years with emotional behavioral problems (EBP). We hypothesize that children with EBP undergoing PCIT will adhere to and tolerate wearables (e.g., Garmin vivosmart4 smartwatch) by having at least 70% of enrolled patients being able to wear it 70% of their treatment period. This will be monitored by checking sleep, heart rate, and intensity of data obtained from Garmin watches. The study will also evaluate the effectiveness of AI-PCIT by comparing weekly behavioral treatment outcomes as compared with control group over a 12-week period. The study will also serve to track indirect behavioral measures on smartwatches (i.e., measurements of heart rate, intensity of activity) from young patients as compared to parental rating scales of ECBI (obtained weekly). These measures can then be used for target engagement biomarkers in future interventional trials. Finally, we will also collect whole blood from both child and parent to investigate exploratory analyses on biomarkers (e.g., C-reactive protein, metabolomics, exposomics) of response to PCIT.

## Methods

Our clinical trial is examining the AI-PCIT for treatment of externalizing behavioral problems in young children. Participants are recruited from Young Child Behavioral Clinic (YCBC) at Mayo Clinic in Rochester, MN, USA, and through advertisements at local pediatrics and family medicine clinics. Interested participants are initially provided information about the study and are scheduled for a consult in the Young Child Behavioral Clinic. The overall recruitment goal for the trial is *N* = 50 participants between ages of 3–7 years whose measure of EBP rated above the clinically significant range (T-score ≥ 60) (Eyberg Child Behavior Inventory-ECBI; Eyberg & Pincus, 1999) [3]. Participants are randomly assigned to AI-PCIT group or PCIT-sham biometric group. Each child is recruited with their parents who are at least 18 years old for a total recruitment of 100 participants. Participants are children aged 3–7 years old. They are of any gender, race, or ethnicity recruited from outpatient or inpatient care. Inclusion criteria include the patients’ ability to provide developmentally appropriate informed assent and legal guardians that are able to provide informed consent. Furthermore, the patient’s EBP severity must be rated above the clinically significant range (≥ 120; T-score ≥ 60) (Eyberg Child Behavior Inventory-ECBI; Eyberg & Pincus, 1999) [3]. The need for more intensive behavioral treatments such as an ER visit or hospitalization for behavioral dyscontrol will not be exclusionary or exit criteria. Participants must be fluent in the English language, have verbal abilities, and have the competency to consent based on ability to provide a spontaneous narrative description of the key elements of the study. Participants also require at least one smartphone and Internet access. Exclusion criteria for the PISTACHIo clinical trial include individuals who are unable to speak and understand English; patients who refuse or withdraw their consent, or those who are unable or unwilling to adhere to study procedures; and patients who do not have a smartphone and access to Internet connection. The study has the approval of the local institutional review board for human subject research protection (Mayo Clinic, Rochester, MN, USA).

### Baseline clinical and functional assessment before PCIT treatment

Caregivers will be interviewed using the Kiddie-Schedule for Affective Disorders and Schizophrenia-Early Childhood (K-SADS-EC) which is administered to assess the child’s psychiatric symptoms and assign DSM-5 diagnoses along with the Preschool Age Psychiatric Assessment (PAPA), a reliable measure of Axis I disorders (and severity) in preschool children, (Egger et al., 1999; Egger et al., 2006) [18, 19]. Children’s Global Assessment Scale (CGAS) is completed by the clinician rater to measure children’s global level of impairment. Parenting Stress Index (PSI) is used to measure the magnitude of stress within the parent–child dyad via caregiver report. Coping with children’s negative emotions (CCNES) is used to assess parental coping styles and strategies in response to children’s expression of negative emotions via caregiver report. To capture a wider range of problematic behaviors, this study uses the Child Behavior Checklist (CBCL). Preschool Feelings Checklist (PFC) is used to quickly screen for the presence of any symptoms of depression in our population. The Pediatric Sleep Questionnaire (PSQ) is used to examine sleep behavior in young children. Trauma Symptom Checklist for Young Children (TSCYC) is used for the assessment of trauma-related symptoms in children ages 3–12. Finally, the Early Childhood Screening Assessment (ECSA) is used to identify very young children (1½–5 years old) who need further emotional or behavioral assessment.

Children and parents (one primary caregiver) who qualify and agree to participate in the study are given vivosmart4 smartwatches during their screening/baseline assessment visit. Two external service providers are used for aggregating Garmin-collected smartwatch data (Fitabase, cloud-based product of Small Steps Labs LLC) and to deliver real-time alerts for parents and collect daily/weekly behavioral functioning of study’s children (mEMA: mobile Ecological Momentary Assessment, a mobile application product of Ilumivu). Deidentified subject identifiers (IDs) are used to create accounts for Fitabase and mEMA, thereby no protected health information (PHI) of the patient is made available to any external data aggregation or alert service providers. Data is collected and stored throughout the study which is then analyzed at study closure. Data from Garmin vivosmart4 smartwatches are automatically synced to Garmin Connect via participant’s smart device (e.g., smartphone, tablets) and to Ilumivu’s mEMA smartphone app. Each update on Garmin Connect updates the data in Fitabase as well. Ilumivu’s mEMA app, available on iOS and Android app stores, serves to trigger assessments in an attempt to foster parenting practices based on child’s physiology. Optional blood tests (only if patients and primary caregivers upon providing consent) are collected for exploratory work and ongoing collaborations in our local lab.

### Study procedures

The PISTACHIo clinical trial utilizes a randomized, double blind, design to compare the active PCIT-AI group and PCIT-sham (treatment as usual) group. The child and primary caretaker are fitted with Garmin watches that are required to be worn for a minimum of 70% of the days during their 12 weeks of treatment. In addition to this, we require the participants to wear the watch for at least 70% of every 24 h per the principal investigator’s discretion. Families are scheduled for PCIT treatment with PCIT-certified therapist in the Young Child Behavioral Clinic. Before the first PCIT treatment session, all participants are randomized into an active versus sham arm of the study. This is done by using a computer-generated list based on a stratified randomization scheme and using a permuted block method with a random number generator. The research assistant, research coordinator, and computer engineer who monitors Garmin apps are the only team members aware of which group the participant belongs to (active vs sham) [20]. Study investigators, clinical/cognitive raters, and participants are blind to the treatment condition. Parents in the AI-PCIT group are receiving targeted messages that instruct them on how to de-escalate their child when the Garmin device recognizes vital signs associated with a child’s tantrum. Parents in the PCIT-sham group receive random messages throughout the day with various relaxation strategies such as reminders to practice deep breathing with their child. The treatment is overseen by board-certified child and adolescent psychiatrist who is a Within-Agency PCIT Trainer.

Any participants withdrawn from the study if they were non-adherent to study procedures or met exclusion criteria at any point after the consent will not be replaced, and their data will not be used for analysis. Additionally, broken watches will be replaced, and therefore, the participant will not be excluded from the study.

**Fig. 1:**
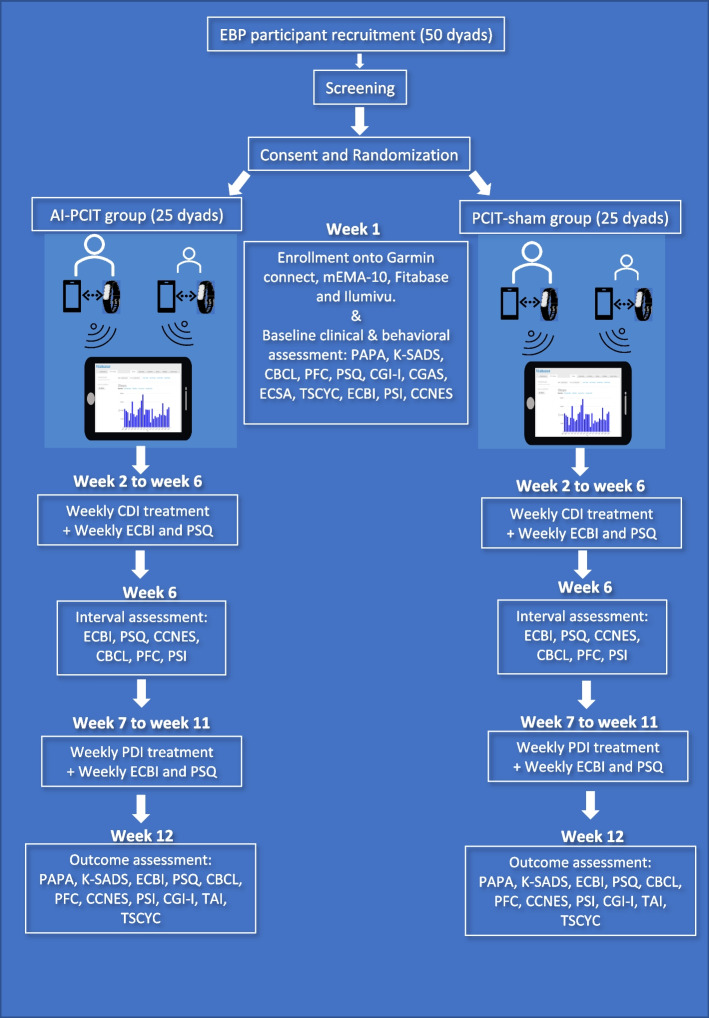
PISTACHIo study workflow

The ongoing clinical trial study protocol conforms to the International Consolidated Standards of Reporting Trials (CONSORT) guidelines and is registered on Oct 14, 2021, in clinicaltrials.gov (ID: NCT05077722), an international clinical trial registry. See Fig. 1 depicting the PISTACHIo study workflow and timing for study procedures.

### Smartwatch technology and momentary assessments

This study will use Garmin vivosmart4 smartwatches (previously used in IRB application 20–002,133 preliminary study) to monitor the step count, sleep quality, heart rate (resting and active), and activity rates (i.e., how often subject moves). These data are available with granularity of each minute and will be recorded through Fitabase for both parents and children. Furthermore, for those in the active PCIT-AI arm, the child’s watch will feed data into mEMA that will trigger a behavioral alert to be sent to the parent participant. Study staff will walk the parents/guardians through how to download the Garmin Connect and mEMA applications on their smartphones or tablets to allow for syncing of the data from the smartwatch to Fitabase or mEMA. Families who do not have their own smartphone or tablet will be provided with an iPod. We note that the choice of iPod for the study was made prior to Apple Inc.’s announcement of discontinuing the product’s manufacturing (May 10, 2022), but the iOS 16 will support the iPods until the study’s anticipated end date. Families will be guided to synchronize data at a minimum once at each PCIT appointment, yet ideally, every night. Garmin watches are waterproof, and participants will be encouraged to wear them at all times during the 12 weeks of treatment (minimum of 70% of the time, 70% of the days). The choice of 70% as a minimum threshold for compliance during the study allows for meaningful imputation of missing values [21]. Acoustic analysis of child vocalizations recorded during sessions will be analyzed using frequency and joint time–frequency modulation methods [22].

### Biological specimen collection for CRP and multi-omics studies

This study will collect 11 mL of blood from both participating subjects (child, parent/significant caregiver) for biomarker discovery efforts. Particularly, this study will use blood to derive C-reactive protein (CRP) levels, genome-wide genomics, proteomics (using Olink® Explore 3072 platform), metabolomics, and exposomics.

CRP is a known marker of chronic inflammation. At high levels, it alerts to acute infection; however, minor elevations can point to stress-related immune dysregulation and has been associated with psychosocial stress [23]. Much is known about effects of elevated CRP levels in adulthood on long-term medical and psychiatric issues (chronic stress); however, little is known about the origins of CRP elevation early in life [24]. To date, there has been only one study that explored CRP in preschoolers that showed no significant correlations between mother’s chronic inflammation and child inflammation [25]. They hypothesized that it might be difficult to detect chronic inflammation in children as young as 3 years old; however, this was only a pilot study. Other studies were able to show strong associations between CRP levels and early childhood stress in children as young as 10 that persisted into adulthood [26].

The exposome is a combination of cumulative environmental influences and associated biological responses throughout the life span, including exposures from the environment, diet, behavior, and endogenous processes [27]. Our prior work with multi-omics has facilitated the discovery of novel biomarkers for studying pathophysiology of disease and/or drug response and the prediction of antidepressant treatment outcomes. Given that the parent–child dyad is characterized by a combination of these factors in addition to inherited factors (i.e., genomics), we hypothesize that integrative analytics of these measures will help identify biomarkers associated with externalizing behavioral outcomes in children. High-resolution mass spectrometry (HRMS) assays will be used to measure the metabolome and exposome.

HRMS analyses follow established protocols using dual liquid chromatography-ultrahigh-resolution mass spectrometry (LC–MS) for metabolomics and gas chromatography-ultrahigh-resolution mass spectrometry (GC–MS) for exposomics with data extraction supporting both targeted and untargeted analyses [28, 29]. Capabilities can identify a very broad spectrum of endogenous metabolites, food and microbiome metabolites, and environmental chemical exposures [28, 29]. The complementary methods detect environmental chemicals, such as PAHs (polycyclic aromatic hydrocarbons), organochlorine pesticides, PCBs (polychlorinated biphenyls), PBBs (phosphate-buffered saline), PBDEs (polybrominated diphenyl ethers) with GC methods, and more polar metabolic products and water-soluble pollutants such as PFAS (per- and polyfluoroalkyl substances) with LC methods [28–30].

Bioinformatic and biostatistical methods are well-developed to detect discriminatory features, metabolites, and environmental chemicals and determine unique exposure patterns such as relative quantities, chemical correlations, and interactive effects of environmental chemicals and metabolic pathway enrichment [31–33]. Approaches using machine learning, mixture modeling, and network analysis tools, such as xMWAS [31, 32], provide powerful approaches for omics data integration, network analysis, and visualization of specific exposome associations to EBP [31–33]. Such exposome and metabolome-wide association studies in these parent–child dyads will link critical exposures and associated metabolic responses to behavioral outcomes. The multi-omics framework will deepen understanding of complex molecular interactions, metabolic perturbations, potential novel biomarkers, and causal mechanisms related to externalizing behavior outcomes.

### Data confidentiality

All nonelectronic questionnaires administered throughout the study will be stored in locked drawers in a secure office. Deidentified data will be entered into a REDCap database that will be protected with passwords available to limited study personnel. Deidentified electronic data from the Garmin smartwatch and mEMA applications will be uploaded on Fitabase and Ilumivu’s password-protected secure websites respectively. Both Fitabase (product of Small Steps LLC) and mEMA (product of Ilumivu) are HIPAA compliant in that no PHI data is needed for data collection and aggregation. Only authorized users will have access to these websites through unique identifiers and passwords. The electronic data will be downloaded to a file available to limited study personnel, and that could only be accessed through Mayo Clinic’s secure intranet on a local computer of the study team.

### Proposed analysis

This study is an exploratory clinical trial with the broad goals of demonstrating feasibility, producing pilot data, generating validate effect sizes, and informing future larger, multicenter trials. The level of significance will be set at *α* = 0.05 (two tailed), and to address multiple testing (where applicable), *p*-values will be adjusted using the false discovery rate. We estimated the sample size for this pilot study based on the hypothesis examining clinical outcome (ECBI) between the PCIT-AI group and PCIT-sham group (hypothesis 2a). We anticipate that a sample size of 50 participants per group (*N* = 100) achieves 70% power, at a 0.05 alpha level (two tailed) to detect an odds ratio of 2.0 favoring PCIT-AI with the proposed randomized design. To evaluate feasibility of the use of Garmin wearables, we will use descriptive statistics assessing the frequency of Garmin use in participants (70% or greater is defined as success for this hypothesis). A linear mixed model analysis of repeated measures will be used to evaluate the interaction effects of type of PCIT (AI informed or AI sham), time, and ECBI scores. A separate linear mixed model analysis will be used to evaluate the interaction effects of PCIT type (AI informed or AI sham), time, and PSQ scores with ECBI scores as a covariate. To evaluate concordance of sleep cycle data from children aged 3–7 as compared to parental rating scales of PSQ, we will use Spearman correlation coefficients and mixed linear regression models. Separate Spearman correlations and a mixed linear regression model will examine the relationship between biometric data from smartwatches (heart rate and movement) with weekly ECBI reports. Partial least-square regression networks will be used to derive multi-omic integration networks, and Spearman correlation will then be used to associate omics with physiological measures from smartwatches.

## Discussion

Currently, there are no AI-augmented treatments for young children with EBP. PCIT is an evidence-based behavioral treatment for 3–7 years old with EBP supported by numerous meta-analyses studies [12, 34, 35]. Attrition rates in PCIT are high and can reach 25 to 69% depending on the study [13, 36]. Factors such as education, income, family employment, and parents’ engagement have been cited as main reasons for attrition [37].

Our study is first to offer AI-augmented PCIT treatment for young children with EBP which will allow for improved engagement of families. The main goal of PCIT is to strengthen the relationship between the parent and child. With the use of our Garmin technology, we can augment that goal by sending targeted messages to parents in critical moments when their child needs help. What is particularly innovative about our project is that the chosen smartwatch does not require the young child to spend time in front of a screen or to interact with an app, both situations that many parents are opposed to for children of this age range. Instead, it helps parents to remember important therapy techniques during real-world parent/child interactions which can be vital learning opportunities for the dyad.

Our study additionally provides us with a valuable environment to test the relationship between multi-omic associations of disruptive behaviors in a parent–child dyad and associations with physiological smartwatch measures. This has never been studied in children with EBP.

It is important to note that data privacy is a significant concern in mobile health, particularly in the research area. As a procedure at enrollment, the study team disengages location, calendar, contacts, social media, and microphone settings on the Garmin Connect and mEMA applications. However, the study will not stop participants from using their discretion to change these settings should they choose to utilize additional features commodity smartwatches provide. Additionally, all self-reported assessments through the mEMA application are deidentified as the connection between the application, and the Ilumivu website is done through a randomly generated code accessed by IRB-approved study personnel.

Another important ethical consideration is related to the burden of the mEMA app. Generally, EMA methods require time commitment on behalf of the patients, and in our study, it relies on daily assessments done on the mEMA app. This could affect the adherence of the participants. However, receiving notifications as daily reminders and reminding patients about the daily assessments every week during therapy should improve compliance. Additionally, participants will receive weekly reimbursement for completing the assessments and therapy sessions which should also cause a significant increment in compliance. The mEMA application is easy to use with a simple interface that directly takes the participants to the assessments. Moreover, because EMA relies on recording participants’ continuous experiences, this may be more invasive than having the participant answers a retrospective questionnaire. This should prompt the consideration of the risk of intrusiveness into the daily life of the patients on one hand and the inconvenience of receiving requests at inopportune times.

The benefit of using commodity smartwatches to predict tantrums lies in the ubiquity of these devices. Approximately 3 in 10 children wear smartwatches, and that number is expected to grow. Valid physiologic smartwatch biomarkers will have a number of clinical and research implications. Smartwatch biomarkers will be readily scalable for widespread clinical practice. Smartwatch physiologic biomarkers will revolutionize clinical trial research in children and adolescents [38–41]. The research team has active plans for future studies to develop diagnostic biosignatures and interventional trials for children with mental health disorders. These future clinical trials through applications to the National Institutes of Health will span novel behavioral interventions, pharmacological agents, and technology-based interventions informed by FDA guidance on “real-world evidence” [38, 42–45].

## Conclusion

The PISTACHIo study is positioned to determine whether AI-PCIT treatment is a superior treatment of EBP. For the first time, we will be able to determine tolerability of smartwatches in children aged 3–7 with EBP. Given the novelty of the study in engaging with parents and children through smartwatches and mobile applications, we anticipate the following findings that will guide future digital health interventions for families with children diagnosed with disruptive behavior. First, we expect that very young children with severe behavioral and compliance issues will want to wear, and tolerate wearing, smartwatches for 12 weeks of the study. Second, we expect that patients commit to daily, albeit brief, engagement with the app and weekly therapy sessions. This intense schedule may limit our ability to recruit to our trial. On the other hand, patients will be encouraged to communicate with research staff in case of issues, and children will be offered toys for their willingness to wear their watch every week. This may cause some unintended therapeutic effects. Typically, in PCIT, children are not provided toys on a weekly basis.

### Clinical significance

The current study aims to develop an innovative wearable tracking protocol that will use AI technology. This research has the potential to begin critical work on innovative assessment strategies for the youngest patients struggling with significant emotional-behavioral issues. Smartwatch data and artificial intelligence approaches will have enhanced temporal resolution compared to standard assessments, again proving the importance of this work. This would be the first time such a treatment model is used in children below 8 years of age, and, as such, our proposal is innovative in a number of ways. These innovations will facilitate the following: (1) collection of more objective and longitudinal behavioral measures; (2) assessments of standard clinical outcome measures such as the ECBI, PSQ, and the rate of tantrum events/duration everyday activities; (3) identification of early opportunities for reinforcing positive behavior that is child-specific; and (4) an understanding of the origins behind childhood disruptive behavior. Artificial intelligence methods such as reinforcement learning and probabilistic graphical models will be used to develop methods for data analysis. These methods are suitable for longitudinal data such as the data gathered in this trial. Information collected from the PISTACHIo research study can incorporate time-sensitive changes as well as physician knowledge to make childhood prognoses detailed and interpretable.

## Data Availability

The datasets used and analyzed during the current study are available from the corresponding author on reasonable request.
